# Chronotype, Night Shift Work, and Diurnal Salivary Cortisol Rhythms Among Healthcare Professionals

**DOI:** 10.3390/jcm14217630

**Published:** 2025-10-28

**Authors:** Katalin Fusz, András Deák, Péter Závodi, Gergely Suszter, Katalin Böröcz, Dávid Szinger, Alain le Roux, Nóra Rozmann, Peter Laszlo Kanizsai

**Affiliations:** 1 Institute of Physiology, Medical School, University of Pécs, 7624 Pécs, Hungary; 2 University Emergency Department, Clinical Centre, University of Pécs, 7624 Pécs, Hungary; deak.andras@pte.hu (A.D.); kanizsai.peter@pte.hu (P.L.K.); 3 Faculty of Pharmacy, University of Pécs, 7624 Pécs, Hungary; 4 Department of Immunology and Biotechnology Medical School, University of Pécs, 7624 Pécs, Hungaryleroux.alain@pte.hu (A.l.R.); 5 Pedagogy of Health and Nursing Sciences, Institute of Emergency Care, Faculty of Health Sciences, University of Pécs, 7621 Pécs, Hungary

**Keywords:** chronotype, shift work, perceived stress, insomnia, cortisol, healthcare professionals

## Abstract

**Background**: Chronotype is a determinant of tolerance to shift work; however, its physiological and psychological correlates remain insufficiently explored in healthcare workers. This study investigated associations between chronotype, perceived stress, sleep quality, and health behaviours in a large cohort of shift-working nurses and physicians. Additionally, diurnal salivary cortisol patterns were characterised in a physiological subsample. **Methods**: A cross-sectional study was conducted with 451 participants (77% female; mean age 42 ± 11 years) completing validated instruments, including the Perceived Stress Scale (PSS), Athens Insomnia Scale (AIS), Patient Health Questionnaire Somatic Symptom Scale (PHQ-15), and reduced Morningness–Eveningness Questionnaire (rMEQ). In addition, a prospective pilot substudy was performed in a physiological subsample of nurses (*n* = 40), in which salivary cortisol was measured at three time points during both day and night shifts. **Results**: Evening chronotype exhibited a higher prevalence of insomnia (70%) and elevated AIS scores (8.2 ± 4.2, *p* < 0.001). In the physiological subsample, evening types demonstrated a significantly attenuated cortisol awakening response (6.5 ± 5.1 nmol/L, *p* = 0.02) and a flatter diurnal cortisol slope during day shifts (*p* = 0.01). Logistic regression indicated that increased age, somatic symptom burden, and perceived stress were significant risk factors for insomnia, whereas morningness was protective (OR = 0.89, *p* = 0.003). **Conclusions**: Evening chronotype among healthcare professionals is associated with altered hypothalamic–pituitary–adrenal axis regulation and impaired sleep quality. These findings highlight the potential utility of chronotype-based scheduling and behavioural interventions targeting circadian misalignment to improve occupational health outcomes in shift-working populations.

## 1. Introduction

Circadian rhythms regulate fundamental biological processes in the human body. These rhythms are generated by internal biological clocks and are synchronised with environmental cycles through key zeitgebers such as light, food intake, physical activity, and social interactions [1]. Central regulation is exerted by the suprachiasmatic nucleus (SCN) of the hypothalamus, which coordinates hormonal and autonomic systems to maintain circadian alignment. When misalignment occurs between internal biological time and the external environment, prolonged disruption may increase the risk of adverse health outcomes [2].

Shift work—particularly night and rotating shifts—is among the most important disruptors of circadian regulation. Such work patterns desynchronise internal rhythms and, in the long term, increase the risk of cardiovascular, metabolic, and mental health problems [3,4]. Previous studies have shown that shift work can heighten stress sensitivity, impair sleep quality, and alter the daily profile of cortisol secretion [4,5,6,7].

Cortisol, the main glucocorticoid hormone produced by the hypothalamic–pituitary–adrenal (HPA) axis, is a key marker of the stress response. Its secretion is under circadian control via SCN-driven neuroendocrine pathways. Cortisol levels normally peak in the early morning—the cortisol awakening response (CAR)—and gradually decline throughout the day [8]. However, this rhythm is not fixed; it is influenced by the sleep–wake cycle, workload, social activity, and individual chronotype [6,9]. CAR can be interpreted as an increased response to potential stressors following awakening, but it is blunted or absent if awakening does not occur in the “correct” circadian phase. This can be especially problematic for shift workers who often wake up at times that are contrary to their internal biological clock, which can result in a “blunted CAR” state. Increased CAR is characteristic of high work stress, and general life stress. Blunted CAR (reduced, “flat” awakening cortisol response) is observed in chronic fatigue, burnout, and post-traumatic stress syndrome (PTSD). Thus, reduced CAR is not necessarily favourable and may indicate hypofunction or adaptive withdrawal of the HPA axis, especially after chronic stressful situations [10,11].

Chronotype refers to inter-individual differences in preferred timing of sleep and activity, classically categorised as morning, intermediate, or evening types. It has a substantial genetic component but is also shaped by environmental and social factors such as light exposure, work schedule, and lifestyle [12]. Chronotype plays a pivotal role in tolerance to shift work [13,14]. Different chronotypes can adapt to different shift types, and this affects sleep quality. Interactions between chronotype and shift type determine sleep parameters: morning types sleep poorly after night shifts; evening types suffer most during day shifts; and intermediate types are the most adaptable [13].

Healthcare professionals are particularly vulnerable, as their work schedules rarely align with their internal biological rhythms. Against this background, the present study aimed to investigate the relationships between chronotype, shift type, and physiological stress responses—with particular focus on salivary cortisol—among healthcare workers. In addition to cortisol, we measured blood pressure and heart rate, and assessed subjective stress sensitivity, psychosomatic complaints, sleep quality, chronotype, and night work characteristics.

## 2. Materials and Methods

### 2.1. Sample and Study Design

**Figure 1:**
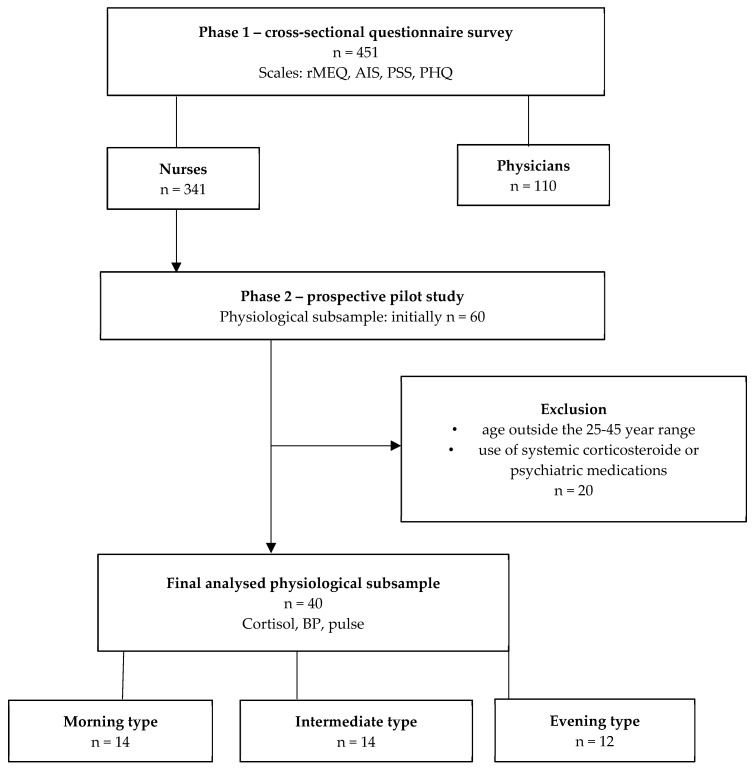
**Flowchart of participant recruitment and analysis. Note:** The figure summarises participant inclusion and exclusion across both study phases. From the initial cohort of 451 healthcare professionals completing the online survey, a subset of 60 volunteers participated in the physiological (salivary cortisol, BP and pulse) substudy. After applying exclusion criteria, 40 participants remained for final analysis. These individuals were categorised into three chronotype groups (morning type: *n* = 14; intermediate type: *n* = 14; evening type: *n* = 12) based on the Morningness–Eveningness Questionnaire (MEQ) scores. Abbreviations: AIS—Athens Insomnia Scale; MSF—midpoint of sleep; PHQ-15—Patient Health Questionnaire Somatic Symptom Scale; PSS—Perceived Stress Scale; rMEQ—reduced Morningness–Eveningness Questionnaire; BP—blood pressure.

This investigation was carried out in two consecutive phases: an online nationwide questionnaire survey among healthcare professionals (nurses and physicians), followed by a prospective pilot study in a physiological subsample of nurses (Figure 1). The study was conducted in accordance with the Helsinki Declaration. and the protocol was approved by the National Medical Research Council Ethics Committee (ETT TUKEB: NNGYK/31233-7/2024).

#### 2.1.1. Survey Sample

Hungarian nurses and physicians participated voluntarily and anonymously in an online questionnaire survey. Eligibility required at least one year of active employment in healthcare. This phase aimed to characterise chronotype distribution, work-related stress, and sleep disturbances in a large professional cohort.

#### 2.1.2. Physiological Subsample

In February 2025, purposive sampling was conducted among nurses employed at the Emergency Department of the Clinical Centre of the University of Pécs. Forty participants were selected and classified into three chronotype groups (morning; intermediate; evening) based on the reduced Morning–Evening Questionnaire (rMEQ) [15]. Participants provided saliva samples and underwent haemodynamic assessments (pulse and blood pressure) at identical time points during both a day shift and a night shift.

The inclusion criteria were as follows. All participants were recruited from the same department, ensuring comparable organisational and environmental conditions. They were engaged in 12 h shifts (day: 07:00–19:00; night: 19:00–07:00) and had been working in rotating shifts for at least one year. To reduce demographic variability, we aimed for a balanced distribution of age (25–45 years) and gender across chronotype groups, insofar as the workforce allowed.

Exclusion criteria were systemic corticosteroid or psychiatric medication use; endocrine disorders (e.g., Cushing’s syndrome, Addison’s disease), and pregnancy.

Saliva samples were collected by participants themselves according to detailed instructions and standardised protocols. While self-collection may raise concerns about adherence, it increases ecological validity by capturing cortisol rhythms in real-life conditions. All participants provided written informed consent.

In view of the known influence of hormonal fluctuations (e.g., menstrual cycle, hormonal contraceptives) on cortisol secretion, additional analyses were restricted to male participants. These revealed similar associations between chronotype and physiological outcomes, suggesting a limited impact of sex hormones. Moreover, oral contraceptive use (*n* = 9) was included as a covariate in sensitivity analyses and did not significantly affect cortisol or related stress biomarkers. Hence, both male and female participants were retained in the final analyses.

### 2.2. Questionnaires

#### 2.2.1. Reduced Morningness–Eveningness Questionnaire (rMEQ)

The validated Hungarian version of the rMEQ was used to assess chronotype, i.e., whether participants had a morning, evening, or intermediate time preference. The questionnaire consists of five items on preferred wake and bedtimes, morning alertness, and peak performance during the day. Scores range from 4 to 25, with values > 16 indicating morning type, <12 evening type, and 13–16 intermediate type [15,16]. Assessing chronotype enables the consideration of individual differences in biological rhythms when examining the impact of shift work on stress responses and circadian misalignment. Internal consistency in our sample was good (Cronbach’s α = 0.80).

#### 2.2.2. Perceived Stress Scale (PSS-14)

The validated Hungarian version of the PSS-14 was used to measure subjective stress perception during the previous four weeks [17,18]. The 14 items assess perceived control, overload, and helplessness in everyday situations, each rated on a 0–4 Likert scale, yielding a total score of 0–56. Higher scores indicate greater stress sensitivity and reduced coping capacity. The PSS provides a general measure of perceived stress, allowing integration of psychological and physiological stress responses. Internal consistency in our sample was excellent (Cronbach’s α = 0.90).

#### 2.2.3. Patient Health Questionnaire Somatic Symptom Scale (PHQ-15)

The validated Hungarian PHQ-15 was applied to assess the frequency and intensity of common somatic symptoms (e.g., headache, fatigue, gastrointestinal or cardiac complaints) in the past weeks [19,20]. Items are scored 0–2, giving a total range of 0–30. Cut-off points of 5, 10, and 15 represent mild, moderate, and severe symptom burden, respectively. As psychosomatic complaints are strongly linked to chronic stress, the PHQ-15 allows examination of the association between subjective well-being and stress regulation. Internal consistency in our sample was good (Cronbach’s α = 0.84).

#### 2.2.4. Athens Insomnia Scale (AIS)

The AIS is an eight-item validated instrument assessing insomnia symptoms and their impact on daytime functioning, based on ICSD diagnostic criteria [21,22,23]. Each item is scored 0–3, with total scores ranging from 0 to 24. A cut-off of ≥6 indicates clinically relevant insomnia. The AIS captures both nocturnal sleep difficulties and daytime consequences, making it suitable for epidemiological and clinical studies of sleep disorders. Internal consistency in our sample was good (Cronbach’s α = 0.83).

#### 2.2.5. Sociodemographic and Work-Related Variables

The self-administered questionnaire collected data on sociodemographic background (age, sex, marital status, children, educational attainment, professional role, hospital department, additional employment, and university studies), health-related behaviours (physical activity, diet, insomnia, self-rated health), and occupational factors (shift schedules, night work frequency and preference, well-being during day and night shifts, symptoms related to night duty, and workplace lighting). These variables provided a comprehensive profile of participants’ personal, lifestyle, and work-related characteristics relevant to stress regulation.

### 2.3. Physiological Data Collection

Salivary cortisol was assessed at three time points: C1 (30 min after awakening; cortisol awakening response, CAR), C2 (18:00), and C3 (22:00). The 0 min post-awakening sample was omitted to reduce participant burden in this working population. The 30-min post-awakening value is widely accepted as a robust marker of CAR, consistent with prior literature [10,11,24,25].

Sampling was performed on two workdays per participant: one day shift and one night shift. Participants self-collected saliva samples following standardised written and verbal instructions, stored them at −20 °C. Salivary cortisol concentrations were determined using a commercially available enzyme-linked immunosorbent assay (ELISA) kit (Cortisol Saliva ELISA, RE52611, IBL International, Hamburg, Germany) following the manufacturer’s instructions. Optical density was read at 450 nm, and cortisol levels were expressed in nmol/L [26].

Reference ranges were based on manufacturer data and large-scale normative studies. For CAR, values below 5.5 nmol/L were categorised as “low morning cortisol” (blunted CAR), following established cut-offs [10,11,25]. Reference ranges for evening cortisol were 2–6 nmol/L at 18:00 and 1–3 nmol/L at 22:00 [27,28].

In addition to cortisol, participants measured blood pressure and heart rate at each sampling point and maintained a logbook recording shift type, sleep–wake times, and subjective stress levels (1–10 scale).

### 2.4. Statistical Analysis

All statistical analyses were performed using IBM SPSS Statistics, version 29.0 (IBM Corp., Armonk, NY, USA). Descriptive statistics were calculated for all variables. Categorical variables were compared using Fisher’s exact test due to small subgroup sizes. Post hoc Bonferroni correction was required when the Chi-square test reached significance. For continuous variables assessed with one-way ANOVA, we performed Levene’s test for homogeneity of variances; if ANOVA F was significant, we applied Bonferroni post hoc pairwise comparisons. For continuous variables that were non-normally distributed (identified by Shapiro–Wilk), we applied Kruskal–Wallis; when Kruskal–Wallis was significant, we carried out pairwise Mann–Whitney U tests with Bonferroni correction (alpha adjusted by number of pairwise comparisons). Paired comparisons (day vs. night shifts) were conducted using the Wilcoxon signed-rank test. Associations between psychological scales (PSS, rMEQ, PHQ-15, AIS) were examined via linear regression analyses. Univariate general linear models (GLMs) were applied to estimate the independent and interaction effects of chronotype, demographics, and psychological variables on cortisol parameters (CAR, area under the curve [AUC], diurnal slope), as well as on PSS, PHQ-15, and AIS scores. To further explore predictors of insomnia (AIS ≥ 6), we used binary logistic regression, including age, somatic symptoms (PHQ-15), perceived stress (PSS), and chronotype as covariates. Missing data were handled using listwise deletion, leading to small variations in sample size across models. Statistical significance was set at *p* < 0.05.

## 3. Results

### 3.1. Survey-Based Assessment of Stress, Sleep, and Health Behaviours in Healthcare Professionals

The sample comprised 451 healthcare professionals, including 341 nurses (76%) and 110 physicians (24%). The average age of the participants was 42 years (SD = 11, range 21–70), and 74.2% were female (*n* = 349). Participants from all counties of Hungary participated in the survey, from a total of 29 types of specialist practices and hospital departments, most of them from emergency departments (*n* = 118, 26%). We assessed several health behaviour variables that may influence stress and sleep quality: 85% of respondents work at night, 39% exercise regularly, 71% consume coffee daily, 8% energy drinks, 6% alcohol several times a week and 3% sleeping pills. Based on the reduced Morningness–Eveningness Questionnaire (rMEQ), 34% of participants were classified as morning types, 53% as intermediate, and 13% as evening types. The proportion of nurses and physicians was similar across chronotype groups (*p* > 0.20).

Marked occupational and health-related differences were observed between the two professional groups. Among nurses the majority (82.3%) worked in rotating or multi-shift schedules, 14.7% worked exclusively day shifts, and 2.9% worked only night shifts. Physicians most frequently reported working day shifts with additional on-call duties (60.6%), followed by 12 h day–night shifts (25.7%). Only 11% worked exclusively daytime schedules, while a minority worked exclusively night shifts (0.9%) or 24-h shifts (1.8%). Nurses reported a significantly higher number of monthly night shifts (M = 5.88 vs. 4.03, *p* < 0.001) and were more frequently scheduled for night duty (M = 5.75 vs. 4.32, *p* < 0.001). They also reported better subjective well-being during night shifts (M = 5.58 vs. 4.33, *p* < 0.001), whereas physicians indicated fewer night-related complaints (M = 1.87 vs. 1.43, *p* = 0.008). Nurses had a higher body mass index (BMI: 27.3 vs. 25.5 kg/m^2^, *p* = 0.002) and reported greater somatic symptom severity (PHQ-15: 9.22 vs. 7.82, *p* = 0.008). In contrast, physicians exhibited higher perceived stress scores (PSS: 26.8 vs. 24.9, *p* = 0.051, trend-level) and demonstrated a later chronotype, both by rMEQ scores (15.23 vs. 15.99, *p* = 0.048) and midpoint of sleep (MSF: 3:56 vs. 3:21, *p* < 0.001). No significant difference was observed in insomnia severity between the groups (AIS: *p* = 0.116).

Chronotype was associated with multiple sociodemographic, occupational, and health-related characteristics. Evening types were the youngest (37.2 ± 8.1 years) compared to intermediate (41.7 ± 9.3 years) and morning types (44.7 ± 10.1 years, *p* < 0.001). Morning types were more likely to live with children (54%), compared with intermediate (42%) and evening types (27%, *p* = 0.001), and were also less frequently married or in a partnership (58%) than intermediate (75%) and evening types (75%, *p* = 0.003). Occupational patterns also differed. Evening types reported the fewest years of experience in their current shift schedule (11.0 years vs. 15.0 and 13.1 years, *p* = 0.04) but the highest number of monthly night shifts (6.3 vs. 4.7 and 5.7, *p* = 0.001). They rated night work as more suitable (6.4 ± 2.0 vs. 4.7 ± 2.4 for morning types, *p* < 0.001) and reported the lowest well-being during day shifts (5.5 ± 2.1 vs. 6.9 ± 2.1, *p* < 0.001), but the highest during night shifts (6.2 ± 1.9 vs. 4.5 ± 2.1, *p* < 0.001). Evening types drank the most energy drinks (17%; *p* < 0.001). Health-related outcomes also varied by chronotype. Evening types had the highest prevalence of insomnia (70% vs. 49% and 55%, *p* = 0.02) and the highest AIS scores (8.2 ± 4.2, *p* < 0.001). Morning types showed the highest BMI (27.9 ± 5.3 kg/m^2^, *p* = 0.02). No significant differences were found in perceived stress (PSS) or somatic symptoms (PHQ-15) across groups (*p* > 0.20). Interestingly, despite working more night shifts, evening types reported the lowest prevalence of symptoms during night shifts (50% vs. 67% and 73%, *p* = 0.02). A detailed summary of the analysis, including post hoc results, is presented in Table 1. A binary logistic regression analysis identified age, somatic symptom severity (PHQ-15), perceived stress (PSS), and chronotype as significant predictors of insomnia (AIS). Higher age (OR = 1.02, *p* = 0.038), higher PHQ-15 scores (OR = 1.23, *p* < 0.001), and higher PSS scores (OR = 1.07, *p* < 0.001) were associated with increased odds of insomnia, whereas higher rMEQ scores (morningness) were protective (OR = 0.89, *p* = 0.003). Sex and educational level were not significant predictors.

In the general linear model predicting somatic symptom severity (PHQ-15), significant predictors included night-shift-related complaints (B = +1.36, 95% CI [1.07, 1.65], *p* < 0.001), perceived stress (PSS; B = +0.17, 95% CI [0.11, 0.22], *p* < 0.001), and insomnia severity (AIS; B = +0.47, 95% CI [0.35, 0.60], *p* < 0.001). Each additional night-shift complaint corresponded to an increase of 1.36 points on the PHQ-15, while higher stress and insomnia scores also contributed substantially to somatic symptom burden. Demographic variables showed weaker or nonsignificant associations: although sex and education reached significance in the overall model, parameter estimates were small and not clinically meaningful (e.g., female sex: B = +0.27, *p* = 0.87; nurse status: B = +0.35, *p* = 0.85). Chronotype (rMEQ, MSF), number of night shifts per month, and lifestyle factors (e.g., physical activity, living with children) were not significant predictors.

The model predicting insomnia severity (AIS) was significant (F = 10.33, *p* < 0.001), explaining 48.4% of the variance (adjusted R^2^ = 0.437). Older age (B = 0.033, *p* = 0.036) and a greater number of night shifts per month (B = 0.151, *p* = 0.021) were associated with higher AIS scores, indicating poorer sleep quality. Psychological and somatic variables emerged as the strongest predictors: higher perceived stress (PSS; B = 0.131, *p* < 0.001) and greater somatic symptom severity (PHQ-15; B = 0.302, *p* < 0.001) were strongly linked to insomnia severity. Chronotype also played a role, with evening orientation (lower rMEQ scores) predicting higher AIS values (B = −0.122, *p* = 0.021). In contrast, sex, parental status, education level, sport activity, midpoint of sleep (MSF), and night-shift-related complaints were not significantly associated with insomnia, and no interaction effects were detected.

The model predicting perceived stress (PSS) was also significant (F = 9.32, *p* < 0.001), accounting for 45.8% of the variance (adjusted R^2^ = 0.409). Both somatic symptom severity (PHQ-15; B = 0.585, *p* < 0.001) and insomnia severity (AIS; B = 0.729, *p* < 0.001) showed strong positive associations with higher stress levels, underscoring their central role in stress vulnerability. Education contributed significantly (F = 6.24, *p* = 0.013), with physicians reporting higher stress scores than nurses. No significant associations were observed for age, sex, chronotype (rMEQ, MSF), night-shift complaints, parental status, or sport activity, and no meaningful interaction effects emerged (Table 2).

### 3.2. Cortisol Profiles and Physiological Stress Markers Across Chronotype Groups in a Subsample of Shift Workers

From the initial cohort of 451 healthcare professionals completing the online survey, a subset of 60 volunteers participated in the physiological (salivary cortisol, BP and pulse) substudy. After applying exclusion criteria, 40 participants remained for final analysis.

Among the 40 participants, the distribution of chronotypes was 35% morning, 35% intermediate, and 30% evening types. The groups did not differ significantly in sex, marital status, or participation inside jobs or university studies (*p* > 0.05 for all). However, morning types were older than both intermediate and evening types (34.6 ± 5.4 vs. 30.4 ± 5.3 and 29.3 ± 3.3 years, *p* = 0.02) and were more likely to live with children (57% vs. 21% and 8%, *p* = 0.02). The participants do not take medication and do not consume alcohol regularly.

With respect to psychological outcomes, evening types reported the highest prevalence of insomnia according to the Athens Insomnia Scale (AIS) (67% vs. 57% and 21%, *p* = 0.04) and demonstrated the longest sleep duration after night shifts (6.0 ± 1.3 h vs. 4.9 ± 1.1 h and 4.5 ± 0.7 h, *p* = 0.007).

Salivary cortisol profiles showed pronounced chronotype differences. During day shifts, evening types exhibited markedly lower cortisol awakening response (CAR) levels compared to morning and intermediate types (6.5 ± 5.1 nmol/L vs. 13.0 ± 10.6 nmol/L and 15.9 ± 10.1 nmol/L, *p* = 0.02), with a higher prevalence of blunted CAR (43% vs. 21% and 14%, *p* = 0.07). Furthermore, morning and intermediate types demonstrated the expected steeper decline in cortisol between the CAR peak and 18:00 (−0.93 ± 0.89 and −1.24 ± 0.85 nmol/L/h), whereas evening types displayed a significantly flatter diurnal slope (−0.35 ± 0.37 nmol/L/h, *p* = 0.01). A similar, albeit non-significant, pattern was observed during night shifts (*p* = 0.23). No significant group differences were observed for BMI, PHQ-15, or PSS (*p* > 0.1). A detailed summary of the analysis, including post hoc results, is presented in Table 3.

**Figure 2:**
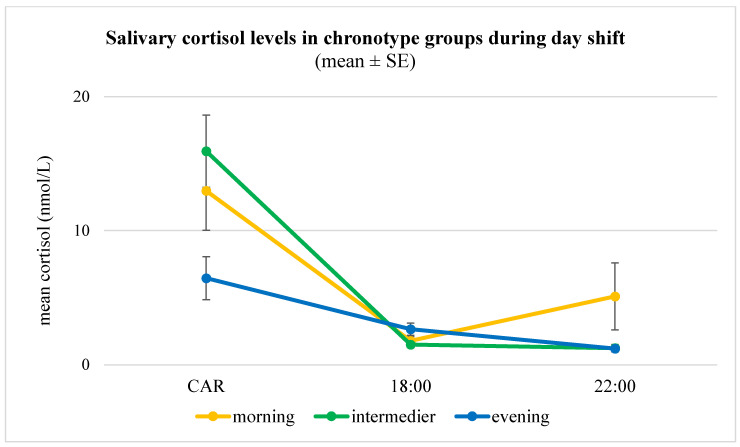
**Salivary cortisol levels (mean ± SE) across chronotype groups during a day shift (*n* = 40). Note:** Concentrations were measured at three time points (30 min post-awakening [CAR], 18:00, and 22:00), revealing distinct diurnal patterns. Morning and intermediate chronotypes showed the expected pronounced CAR followed by a steep decline, consistent with a typical circadian rhythm. In contrast, evening chronotypes exhibited a blunted CAR and a flatter overall slope, suggesting attenuated HPA axis activation and altered diurnal cortisol regulation. Error bars indicate standard errors of the mean (SE). A univariate general linear model confirmed a significant main effect of chronotype on daytime cortisol slope (F(2,24) = 7.99, *p* = 0.002), independent of age, sex, night shift frequency, and psychological measures.

To account for covariates, we performed univariate general linear models (GLMs). The model for CAR accounted for 28% of the variance (adjusted R^2^ = 0.28) and revealed a significant main effect of chronotype (F(2,24) = 7.17, *p* = 0.004), while none of the covariates (sex, age, number of night shifts, PSS, PHQ-15) were significant (all *p* > 0.5). The GLM for daytime cortisol slope was also significant (F(9,24) = 2.53, *p* = 0.033, adjusted R^2^ = 0.30), with chronotype exerting a robust main effect (F(2,24) = 7.99, *p* = 0.002): evening types exhibited a markedly flatter slope compared to morning and intermediate types, independent of covariates (Figure 2).

## 4. Discussion

Our study analysed the relationship between chronotype, work schedule, physiological stress responses, and insomnia among healthcare professionals, particularly those in emergency care, using two complementary approaches. In our previous research, we linked insomnia to increased stress sensitivity and adverse health outcomes, including weight gain and reduced psychological coherence [7]. In a cohort study of Hungarian nurses, mean perceived stress scores (PSS) were like those observed in our current sample [29]. Furthermore, in surveys of healthy adult samples, participants achieved similar scores on the PSS and PHQ-15, but lower scores on the AIS, indicating better sleep quality compared to the healthcare professionals in our study [17,30,31,32]. This underlines that sleep disturbances may be a particularly salient burden of healthcare-related shift work, even when perceived stress levels are comparable to those in the general population.

In our large-scale survey (*n* = 451), intermediate chronotypes predominated, while evening-type participants were younger, more frequently scheduled for night shifts, and more likely to prefer nocturnal work. These findings are consistent with recent evidence showing that morning types are typically older, female, married, and more likely to live with children compared to evening types [33]. Insomnia was most prevalent in evening chronotypes, aligning with previous studies highlighting their vulnerability to circadian misalignment and sleep disorders [9,12].

Independent of chronotype, our findings are consistent with evidence showing that insomnia is multifactorially determined, with age, stress, and somatic symptom burden representing robust predictors in the general population. Epidemiological studies have demonstrated that advancing age is associated with lighter, more fragmented sleep and increased insomnia prevalence [34]. Similarly, perceived stress has been shown to directly predict both sleep onset and maintenance difficulties through hyperarousal mechanisms and maladaptive coping responses [35]. Elevated PHQ-15 scores, reflecting higher somatic symptom reporting, have also been linked to disturbed sleep in population-based samples, suggesting that bodily discomfort and heightened interoceptive sensitivity contribute to insomnia risk [31]. Taken together, these findings support that insomnia arises from an interplay of psychological, physiological, and behavioural factors that extend beyond chronotype-related vulnerability.

The physiological substudy (*n* = 40) provided novel insights into cortisol dynamics. Evening chronotypes exhibited a blunted cortisol awakening response (CAR) and flatter diurnal cortisol slopes, especially during day shifts, suggesting impaired hypothalamic–pituitary–adrenal (HPA) axis activation. While evening types in our study reported longer post-night-shift sleep duration—potentially reflecting both better acute tolerance of nocturnal work and compensatory mechanisms to recover from accumulated sleep debt—they simultaneously showed higher insomnia scores and a greater prevalence of blunted CAR. This paradox supports the notion that chronic circadian misalignment combined with cumulative stress may gradually impair HPA axis regulation [10,25].

Furthermore, the physiological significance of the circadian cortisol rhythm extends beyond occupational health. In individuals with adrenal insufficiency who require glucocorticoid replacement therapy, maintaining a physiological diurnal pattern of cortisol secretion remains one of the most challenging aspects of treatment. Conventional replacement regimens often fail to reproduce the fine-tuned circadian variation in endogenous cortisol secretion, particularly during nighttime or rotating shift work, which may further exacerbate circadian misalignment and metabolic risk [36].

From an occupational health perspective, our findings emphasise chronotype as a potential biological marker of vulnerability among shift-working healthcare professionals. Flexible, chronotype-informed scheduling appears to be the most feasible and health-conscious approach in healthcare settings. Morning-type individuals may benefit from minimising night shift exposure, while frequent night work is also not recommended for evening types due to the cumulative physiological burden and higher risk of health consequences related to circadian rhythm disruption. Therefore, rather than reinforcing extreme eveningness, occupational strategies should support gradual adaptation toward intermediate sleep–wake timing through consistent shift planning, exposure to morning light, and behavioral interventions. This flexibility may optimise both worker well-being and operational efficiency in rotating-shift environments [7,37]. In practice, strict chronotherapy protocols (e.g., structured phase shifts) are rarely feasible in rotating schedules, particularly in emergency care. Instead, lighter behavioural countermeasures are likely to be more practical. Evidence supports the efficacy of bright light therapy in reducing sleepiness and promoting circadian adjustment [38], as well as broader non-pharmacological strategies such as timed light exposure, naps, and structured sleep hygiene [39,40,41]. Taken together, these results suggest that pragmatic, chronotype-informed approaches could mitigate circadian disruption and improve resilience in shift-working populations.

Strengths and limitations. The strengths of this study include its two-phase design that combined a large-scale survey with physiological measurements, thereby integrating self-reported and objective indicators of stress and sleep. This mixed-methods approach enhances the ecological validity of the findings and provides a more comprehensive understanding of the impact of chronotype on shift-working healthcare professionals. This study has some limitations. First, the sample was not representative, with an overrepresentation of nurses and women. Second, although participants received standardised training and instructions, salivary cortisol samples were self-administered during shifts, limiting control over environmental and behavioural factors (e.g., timing, light exposure, pre-sampling activities). Third, menstrual cycle phase and hormonal contraceptive use were not systematically assessed, which may contribute to variability in cortisol levels; however, analyses restricted to men confirmed the robustness of the main findings. Finally, missing data were present both in the questionnaire (average of six missing responses per 5 items) and in the cortisol sub study (23 of 240 samples missing), although the overall proportion remained low.

## 5. Conclusions

Our findings indicate that chronotype significantly shapes both physiological and psychological stress responses among healthcare professionals exposed to shift schedules. Evening chronotypes reported poorer sleep quality and higher insomnia prevalence, together with blunted cortisol awakening responses and flatter diurnal cortisol slopes compared to morning and intermediate types. These alterations reflect dysregulation of the HPA axis and may underlie elevated risks of sleep, mental health, and cardiometabolic disturbances in this vulnerable occupational group.

Our findings underscore the importance of incorporating chronotype into occupational health strategies. While strict chronotherapy is impractical in real-world healthcare settings, lighter behavioural interventions—including optimised sleep hygiene, scheduled light exposure, and strategic rest opportunities—offer feasible, evidence-based tools to reduce circadian misalignment. Future longitudinal and interventional studies are needed to test the effectiveness of such chronotype-based interventions and to guide the development of tailored occupational health policies.

## Figures and Tables

**Table 1:** **Sociodemographic, occupational, and psychological characteristics of participants across chronotype groups (*n* = 451). Note:** Data are presented as mean (minimum–maximum) or mean ± SD for continuous variables and *n* (%) for categorical variables. Group differences were assessed using ANOVA or Kruskal–Wallis tests for continuous variables (depending on normality) and χ^2^ or Fisher’s exact tests for categorical variables. Missing data were handled by listwise deletion for each analysis; thus, sample size varies slightly across variables. Post hoc Bonferroni-adjusted pairwise comparisons: ^a^ = significantly different from morning type; ^b^ = significantly different from intermediate type; ^c^ = significantly different from evening type. Abbreviations: AIS—Athens Insomnia Scale; MSF—midpoint of sleep; PHQ-15—Patient Health Questionnaire Somatic Symptom Scale; PSS—Perceived Stress Scale; rMEQ—reduced Morningness–Eveningness Questionnaire.

Sociodemographic, Occupational, and Psychological Characteristics of Participants Across Chronotype Groups (*n* = 451)				
	**Morning**	**Intermediate**	**Evening**	***p* Value**
**Sociodemographic characteristics**				
	*n* (%)			
Participants	151 (34%)	240 (53%)	60 (13%)	-
Sex, female	123 (86%)	183 (80%)	43 (75%)	0.15
Marital status, married/partnership	113 (58%) ^b,c^	180 (75%)^a^	45 (75%) ^a^	**0.003**
Living together with children	82 (54%) ^c^	100 (42%)	16 (27%) ^a^	**0.001**
Degree, nurse	119 (79%)	182 (76%)	40 (67%)	0.18
Hospital ward, emergency department	37 (41%)	57 (25%)	24 (41%)	0.07
Having side job	26 (17%)	50 (21%)	12 (20%)	0.45
Studying at university (nurse)	21 (18%)	37 (20%)	10 (25%)	0.62
	mean (min-max)			
Age (year)	44.7 (22–70) ^b,c^	41.7 (21–66) ^a,c^	37.2 (25–65) ^a,b^	**<0.001**
**Health behaviours and health status**				
	*n* (%)			
Healthy eating	1 (2%)	36 (15%)	24 (16%)	0.051
Regular exercise	22 (37%)	88 (37%)	64 (43%)	0.59
Energy drink consumption, everyday	5 (3%) ^c^	21 (9%)	10 (17%) ^a^	**<0.001**
Insomnia (based on AIS)	74 (49%)^c^	132 (55%)	42 (70%) ^a^	**0.02**
Subjective health status, bad	14 (9%)	20 (8%)	9 (15%)	0.34
**Work schedule characteristics**				
	mean (min-max)			
Years in this shift type	15.0 (1–42) ^c^	13.1 (1–40	11.0 (1–40) ^a^	**0.04**
Night shifts per month	4.7 (0–16.5) ^c^	5.7 (0–18)	6.3 (0–17) ^a^	**0.001**
Perceived suitability of night shifts (1–10 scale)	4.7 (1–10) ^b,c^	5.6 (1–10) ^a^	6.4 (1–10) ^a^	**<0.001**
Well-being during day shifts (point)	6.9 (1–10) ^b,c^	6.3 (1–10) ^a,c^	5.5 (1–9) ^a,b^	**<0.001**
Well-being during night shifts (point)	4.5 (1–10) ^b,c^	5.5 (1–10) ^a^	6.2 (1–10) ^a^	**<0.001**
Mean number of symptoms reported per night shift	1.7 (0–7)	1.5 (0–5)	1.2 (0–6)	0.07
	*n* (%)			
Working at night as well	113 (75%) ^b,c^	218 (91%)^a^	54 (90%) ^a^	**<0.001**
Day shifts with on-call duties (physician)	15 (47%)	40 (70%)	11 (55%)	0.25
Multi-shift work schedule (nurse)	84 (72%) ^b,c^	158 (87%) ^a^	37 (93%) ^a^	**<0.001**
Flexible work schedule (nurse)	59 (54%)	113 (63%)	23 (58%)	0.38
Having symptoms during night shifts	101 (67%)	174 (73%)^c^	30 (50%) ^b^	**0.02**
No preference for night work	78 (52%) ^c^	89 (37%)	18 (30%) ^a^	**<0.001**
Night illumination, adequate	75 (66%)	146 (68%)	39 (72%)	0.60
**Chronotype and psychological measures**				
	mean (min-max)			
rMEQ (point)	19.6 (18–24) ^b,c^	14.9 (12–17) ^a,c^	10.1(6–11) ^a,b^	**<0.001**
MSF (hh:mm)	2:45 (1:15–3:00) ^b,c^	3:38 (3:00–5:00) ^a,c^	4:47 (4:30–7:30) ^a,b^	**<0.001**
	mean (±SD)			
BMI (kg/m^2^)	27.9 (±5.3) ^b^	26.4 (±5.3) ^a^	26.1 (±6.1)	**0.02**
PHQ-15 (point)	8.3 (5.4)	9.1 (5.5)	9.3 (6.2)	0.29
PSS (point)	25.1 (8.9)	25.5 (8.5)	25.6 (10.0)	0.91
AIS (point)	5.8 (3.7) ^c^	6.3 (3.9) ^c^	8.2 (4.2) ^a,b^	**<0.001**
Sleep duration after day shift (hour)	7.1 (1.4)	7.3 (1.4)	7.5 (1.8)	0.17
Sleep duration after night shift (hour)	4.6 (2.0)	4.9 (2.2)	5.2 (1.9)	0.25

**Table 2:** **Predictors of somatic symptoms (PHQ-15), insomnia severity (AIS), and perceived stress (PSS) in a general linear model (*n* = 451). Note:** Values are unstandardised regression coefficients (B) with 95% confidence intervals and *p* values. “n.s.” denotes non-significant predictors. Dashes (—) indicate that the respective variable was the outcome and therefore not included as a predictor. Models were adjusted for sociodemographic and occupational covariates; only significant predictors are displayed. Abbreviations: AIS—Athens Insomnia Scale; PHQ-15—Patient Health Questionnaire Somatic Symptom Scale; PSS—Perceived Stress Scale; rMEQ—reduced Morningness–Eveningness Questionnaire.

Predictors of Somatic Symptoms, Insomnia, and Perceived Stress in a General Linear Model (*n* = 451)			
	**PHQ-15**	**AIS**	**PSS**
Predictor	B, (95% CI), *p*		
Age	n.s.	+0.033 (0.002–0.063), *p* = 0.036	n.s.
Night shifts/month	n.s.	+0.151 (0.023–0.279), *p* = 0.021	n.s.
Night complaints	+1.360 (1.072–1.648), *p* < 0.001	n.s.	n.s.
rMEQ	n.s.	−0.122 (−0.225–0.018), *p* = 0.021	n.s.
PHQ-15	—	+0.302 (0.222–0.382), *p* < 0.001	+0.585 (0.392–0.779), *p* < 0.001
AIS	+0.472 (0.347–0.597), *p* < 0.001	—	+0.729 (0.487–0.971), *p* < 0.001
PSS	+0.165 (0.111–0.220), *p* < 0.001	+0.131 (0.088–0.175), *p* < 0.001	**—**

**Table 3:** **Sociodemographic, occupational, and psychological characteristics of participants across chronotype groups (*n* = 40). Note:** Data are presented as mean (minimum–maximum) or mean ± SD for continuous variables and *n* (%) for categorical variables. Group differences were assessed using ANOVA or Kruskal–Wallis tests for continuous variables (depending on normality) and χ^2^ or Fisher’s exact tests for categorical variables. Post hoc Bonferroni-adjusted pairwise comparisons: a = significantly different from morning type; b = significantly different from intermediate type; c = significantly different from evening type. Missing data were handled by listwise deletion for each analysis; thus, sample size varies slightly across variables. Abbreviations: AIS—Athens Insomnia Scale; AUC—area under the curve; CAR—cortisol awakening response; MSF—midpoint of sleep; PHQ-15—Patient Health Questionnaire Somatic Symptom Scale; PSS—Perceived Stress Scale; rMEQ—reduced Morningness–Eveningness Questionnaire.

Sociodemographic, Behavioural, and Physiological Characteristics of Participants by Chronotype Group (*n* = 40)				
	**Morning**	**Intermediate**	**Evening**	***p* Value**
**Sociodemographic characteristics**				
	*n* (%)			
Participants	14 (35%)	14 (35%)	12 (30%)	-
Sex, female	9 (64%)	7 (50%)	7 (58%)	0.59
Marital status, married/partnership	10 (71%)	10 (71%)	7 (58%)	0.53
Living together with children	8 (57%) ^c^	3 (21%)	1 (8%) ^a^	**0.02**
Having side job	3 (21%)	2 (14%)	1 (8%)	0.66
Studying at university	2 (14%)	3 (21%)	2 (16%)	0.83
	mean (min-max)			
Age (year)	34.6 (26–45) ^c^	30.4 (25–44)	29.3 (25–36) ^a^	**0.02**
**Health behaviours and health status**				
	*n* (%)			
Healthy eating	1 (7%)	4 (29%)	1 (8%)	0.46
Regular exercise	7 (50%)	9 (64%)	5 (42%)	0.41
Coffee and/or energy drink consumption, 3 or more times a day	4 (29%)	6 (43%)	5 (42%)	0.77
Insomnia (based on AIS)	3 (21%) ^c^	8 (57%)	8 (67%) ^a^	**0.04**
Subjective health status, good	7 (50%)	7 (50%)	4 (32%)	0.28
**Work schedule characteristics**				
	mean (min-max)			
Work years in this shift type (year)	8.9 (2–25)	6.5 (1–20)	5,8 (2–10)	0.22
Night shifts per month	7.1 (5.5–12.0)	6.7 (4.0–8.5)	7.0 (6.0–8.0)	0.77
Perceived suitability of night shifts (1–10 scale)	6.7 (4–10)	7.5 (3–10)	8.2 (6–10)	0.14
Well-being during day shifts (point)	7.3 (5–9)	7.3 (4–9)	5.9 (3–8)	**0.05**
Well-being during night shifts (point)	6.1 (3–10)	7.3 (3–10)	7.4 (5–10)	0.17
Mean number of symptoms reported per night shift	0.7 (0–2)	0.6 (0–3)	0	**0.03**
	*n* (%)			
Presence of symptoms during night shifts (% reporting ≥1 symptom)	7 (50%)	4 (29%)	0	**0.01**
Night illumination, adequate	7 (50%)	10 (71%)	7 (58%)	0.15
**Psychological and physiological parameters**				
	mean (min-max)			
rMEQ (point)	18.4 (18–20) ^b,c^	13.8 (12–17) ^a,c^	9.8 (9–11) ^a,b^	**<0.001**
Mid-sleep on free days (MSF, hh:mm)	2:20 (1:15–3:00) ^b,c^	3:54 (3:00–5:00) ^a,c^	5:20 (5:00–7:30) ^a,b^	**<0.001**
	mean (±SD)			
Cortisol awakening response (CAR) during day shift (nmol/L)	13.0 (±10.6)	15.9 (±10.1) ^c^	6.5 (±5.1) ^b^	**0.02**
Cortisol awakening response (CAR) during night shift (nmol/L)	7.1 (±3.2)	13.3 (±7.6)	7.9 (±6.7)	0.09
Cortisol slope between CAR and 18:00 during day shift (nmol/L/h)	−0.9 (±0.9)	−1.2 (±0.8) ^c^	−0.4 (±0.4) ^b^	**0.01**
Cortisol slope between CAR and 18:00 during night shift (nmol/L/h)	−0.4 (±0.3)	−0.9 (±0.7)	−0.3 (±0.3)	0.23
AUC during day shift (nmol/L* 16 h)	89.9 (±55.4)	110.0 (±62.3)	63.2 (±41.0)	0.18
AUC during night shift (nmol/L* 16 h)	65.8 (±23.2)	102.5 (±48.0)	71.1 (±39.9)	0.10
Systolic blood pressure at 18:00 during day shift	119.3 (±12.9)	121.4 (±11.4)	123.6 (±13.6)	0.69
Diastolic pressure at 18:00 during day shift	78.3 (±8.9)	78.2 (±8.1)	75.6 (±8.6)	0.68
Heart rate at 18:00 during day shift	78.9 (±9.7)	78.0 (±16.6)	84.5 (±11.6)	0.41
BMI (kg/m^2^)	25.5 (±5.0)	24.0 (±2.1)	27.2 (±6.7)	0.27
PHQ-15 (point)	4.3 (±2.7)	6.3 (±4.6)	6.1 (±3.4)	0.30
PSS (point)	17.1 (±4.7)	22.2 (±8.8)	21.2 (±6.2)	0.13
AIS (point)	3.6 (±1.9) ^c^	6.4 (±3.7)	6.7 (±2.7) ^a^	**0.02**
Sleep duration after day shift (hour)	8.0 (±1.5)	7.0 (±1.7)	7.5 (±2.0)	0.47
Sleep duration after night shift (hour)	4.5 (±0.7) ^c^	4.9 (±1.1)	6.0 (±1.3) ^a^	**0.007**

## Data Availability

The original contributions presented in this study are included in the article. Further inquiries can be directed to the corresponding author(s).

## Abbreviations

The following abbreviations are used in this manuscript:

AIS	Athens Insomnia Scale
AUC	area under the curve
BP	blood pressure
CAR	cortisol awakening response
GLMs	general linear models
HPA	hypothalamic–pituitary–adrenal
MSF	midpoint of sleep
PHQ-15	Patient Health Questionnaire Somatic Symptom Scale
PSS, PSS-14	Perceived Stress Scale
PTSD	post-traumatic stress syndrome
rMEQ	reduced Morningness–Eveningness Questionnaire
SCN	Suprachiamsatic Nucleus
